# Genome-Wide Association Study for Reproductive Traits in a Duroc Pig Population

**DOI:** 10.3390/ani9100732

**Published:** 2019-09-26

**Authors:** Zhe Zhang, Zitao Chen, Shaopan Ye, Yingting He, Shuwen Huang, Xiaolong Yuan, Zanmou Chen, Hao Zhang, Jiaqi Li

**Affiliations:** National Engineering Research Center for Breeding Swine Industry, and Guangdong Provincial Key Lab of Agro-Animal Genomics and Molecular Breeding, College of Animal Science, South China Agricultural University, Guangzhou 510642, China

**Keywords:** Duroc, pig, GWAS, reproductive traits

## Abstract

**Simple Summary:**

Reproductive traits are economically important in the pig industry, and it is critical to explore their underlying genetic architecture. Hence, four reproductive traits, including litter size at birth (LSB), litter weight at birth (LWB), litter size at weaning (LSW), and litter weight at weaning (LWW), were examined. Through a genome-wide association study in a Duroc pig herd, several candidate single-nucleotide polymorphisms (SNPs) and genes were found potentially associated with the traits of interest. These findings help to understand the genetic basis of porcine reproductive traits and could be applied in pig breeding programs.

**Abstract:**

In the pig industry, reproductive traits constantly influence the production efficiency. To identify markers and candidate genes underlying porcine reproductive traits, a genome-wide association study (GWAS) was performed in a Duroc pig population. In total, 1067 pigs were genotyped using single-nucleotide polymorphism (SNP) chips, and four reproductive traits, including litter size at birth (LSB), litter weight at birth (LWB), litter size at weaning (LSW), and litter weight at weaning (LWW), were examined. The results showed that 20 potential SNPs reached the level of suggestive significance and were associated with these traits of interest. Several important candidate genes, including *TXN2*, *KCNA1*, *ENSSSCG00000003546*, *ZDHHC18*, *MAP2K6*, *BICC1*, *FAM135B*, *EPHB2*, *SEMA4D*, *ST3GAL1*, *KCTD3*, *FAM110A*, *TMEM132D*, *TBX3*, and *FAM110A*, were identified and might compose the underlying genetic architecture of porcine reproductive traits. These findings help to understand the genetic basis of porcine reproductive traits and provide important information for molecular breeding in pigs.

## 1. Introduction

Animal reproductive traits are economically important but are mostly sex-specific (such as sperm quality in males and fertility in females), and most of them are complex and present low heritability. Hence, the genetic improvement on these traits is especially difficult compared to other complex traits in livestock breeding practices. In the pig industry, litter traits are extremely important economic traits for pig production, as they are directly related to production efficiency. Improving the litter size is the main breeding goal and has been intensively selected in breeding programs for decades in many well-organized breeding systems, such as at the Canadian Center for Swine Improvement (http://www.ccsi.ca/). Though genetic gain for these traits has been obtained with traditional breeding strategies, the slow genetic improvement has increased the need for molecular breeding, such as genomic selection [1]. 

The fast development of molecular quantitative genetics methods and high-throughput genotyping techniques has increased the feasibility of genetic improvements of reproductive traits via marker-assisted selection or genomic selection. In genomic selection, it has been proved that the genetic gain could be achieved by incorporating prior information such as candidate genes or quantitative trait loci (QTL) affecting the traits under consideration [2]. Hence, for a better genetic dissertation and breeding practice, it is critical to explore the underlying genetic architecture of reproductive traits. A powerful way consists in testing the association between reproductive records and genetic markers covering the whole genome via genome-wide association studies (GWAS) [3]. 

In the past decade, GWAS was widely used to dissect the genetic architecture of growth [4,5], reproduction [6,7], and meat quality [8,9] traits in a variety of pig populations. These GWAS and former QTL mapping studies together identified 28,720 QTLs in total, of which 2129 QTLs are associated with reproductive traits [10]. However, a limited number of genes were reported for each reproductive trait, which can explain only a small proportion of genetic variance [11]. More QTLs or genes underlying reproductive traits are yet to be uncovered. 

The main objective of this study was to perform a GWAS to identify potentially important single-nucleotide polymorphism (SNPs) or QTL regions associated with four reproductive traits, including litter size at birth (LSB), litter weight at birth (LWB), litter size at weaning (LSW), and litter weight at weaning (LWW) in a Duroc pig population. Subsequently, the potential function of significant chromosomal regions was analyzed in detail. 

## 2. Material and Methods

### 2.1. Ethics Statement

Animal care and experiments were conducted according to the Regulations for the Administration of Affairs Concerning Experimental Animals (Ministry of Science and Technology, China, revised in June 2004) and were approved by the Animal Care and Use Committee of the South China Agricultural University, Guangzhou, Guangdong, China (permit number: SCAU#2013-10).

### 2.2. Population and Phenotyping

The population used in the present study was normally maintained in a breeding herd in Fujian, China. Four reproductive traits, including LSB, LWB, LSW, and LWW, were recorded for all sows in this herd. LSB and LWB were measured within 24 hours after delivery, and LSW and LWW were recorded 24 days (the day of weaning) after delivery. At present, information for 4539 Duroc pigs with 15,662 farrowing records has been collected for a period of 10 years (2008 to 2017). A multi-traits animal model was used to estimate covariance components for calculating heritability and genetic correlation. The models used were as follows:

(1) Y=Xb+Za+Wpe+e

where Y is the vector of phenotypic records, b is the vector of fixed effects including parity and year-season, a is the vector of additive genetic, pe is the vector of permanent environmental effects, e is a vector of residuals, and X, Z, and W are incidence matrices for b, a, and pe. 

Estimated breeding values (EBVs) of all pigs and the reliabilities of EBVs were imputed using animal model best linear unbiased prediction [12] and obtained from the in-farm genetic evaluation software Herdsman swine management platform (S & S Programming, Lafayette, IN, USA). As EBVs include pedigree information, which could be significantly associated with the examined traits rather than with the phenotype, de-regressed EBV (DEBV) were calculated for each pig to remove the contribution of information from relatives. The equation for DEBV [13] is as follows:

(2) $$\mathrm{DEBV}=\mathrm{PA}+\frac{\mathrm{EBV}-\mathrm{PA}}{\mathrm{REL}}$$

where DEBV is de-regressed EBV, PA represents parental average, EBV and REL are the estimated breeding value and reliability of each pig. 

### 2.3. Genomic DNA Extraction and Genotyping

In the present study, a total of 1067 Duroc pigs (81 boars and 986 sows) were genotyped for further GWAS analysis. Genomic DNA was extracted from pig ear tissue using the TaKaRa MiniBEST Universal Genomic DNA Extraction Kit (Ver 4.0). The A_260/280_ ratios of DNA samples were determined with NanoDrop 2000 (Thermo Scientific). The samples with A_260/280_ ratio between 1.7 and 2.0 were genotyped using either the Illumina PorcineSNP60 BeadChip (Illumina, San Diego, CA, USA) or the GeneSeek GGP-Porcine chip (Neogen Corporation, Lansing, MI, USA). 

### 2.4. Data Quality Control

For phenotypes, descriptive statistical analyses were performed in R [14] to check the data quality. For genotypes, quality control on genotypes was performed using the PLINK software [15]. In the present study, the SNPs common between the Illumina PorcineSNP60 BeadChip and the GeneSeek GGP-Porcine chip were retained, and these SNPs were filtered according to the following criteria: (1) call rate <90%; (2) minor allele frequency (MAF) <1%; and (3) Hardy–Weinberg equilibrium (HWE) testing with a *p*-value < 1.00e-6. Following the quality control, 32,147 SNPs were retained for further analysis. In order to check whether population stratification existed in the Duroc pig herd, the genomic kinship between all pairs of individuals was calculated using SNPs ion autosomes. 

### 2.5. Statistical Analysis

The association between each SNP marker and the phenotypes was tested with a single-marker regression mixed linear model by GEMMA software [16]. The statistical model is as follow:

(3) $$y=\mu +\mathrm{Zu}+e;u\sim N\left(0,\;G\sigma_{\alpha }^{2}\right);e\sim N\left(0,\;I\sigma_{e}^{2}\right)$$

where y is a vector of dependent variable (DEBVs in this study), μ is the overall mean, G is the realized relationship matrix constructed with markers, $\sigma_{\alpha }^{2}$ is the additive genetic variance, Z represents incidence matrices corresponding to u, e is the vector of residual errors, and $\sigma_{e}^{2}$ is the residual variance.

To confirm the thresholds for the genome-wide significance and suggestive significance, effectively independent tests based on the independent markers and linkage disequilibrium (LD) block (defined as a set of SNPs with pairwise r square values >0.40) were calculated as in [17]. A total of 9266 effectively independent tests was suggested in the present study. Therefore, the genome-wide significance threshold was 0.05/9266 = 5.40 × 10^−^^6^, and the genome-wide suggestive significance threshold was 1/9266 = 1.08 × 10^−4^. 

### 2.6. Identification of Candidate Genes

Candidate genes were identified according to their physical positions and functions based on the *Sus scrofa* 10.2 reference genome assembly. The SNP-containing or nearest annotated genes for each potential SNP were obtained from the Ensembl release 89 (http://may2017.archive.ensembl.org/index.html) and taken as candidate genes. 

### 2.7. Functional Enrichment Analysis

Genes that were less than 1 Mb away from the potential SNPs were selected and identified as the functional genes. Further Gene Ontology (GO) terms annotation was conducted using the Database for Annotation, Visualization, and Integrated Discovery (DAVID) Version 6.8 [18], then the biological process GO terms were selected with the Benjamini–Hochberg method, adjusted *p*-value <0.05. 

## 3. Results

### 3.1. Description of Phenotypes and Genotypes

Descriptive statistics of DEBVs for the traits of LSB, LWB, LWW, and LSW analyzed in this study are presented in Table 1. The genetic correlations for all pairs of traits are given in Table 2. In the present study, the heritability of LSB, LWB, LWW, and LSW was 0.158, 0.161, 0.173, and 0.140, respectively. The traits of LSB and LWB were strongly and positively correlated, and the correlation between LSW and LWW showed the same pattern. 

There were 38,544 SNPs before frequency and genotyping pruning. Through quality control, 130 and 6267 SNPs were excluded from our dataset due to HWE testing and MAF, respectively. Finally, 32,147 SNPs from 1067 animals were retained for further analysis. 

### 3.2. Genome-Wide Association Results

**Figure 1:**
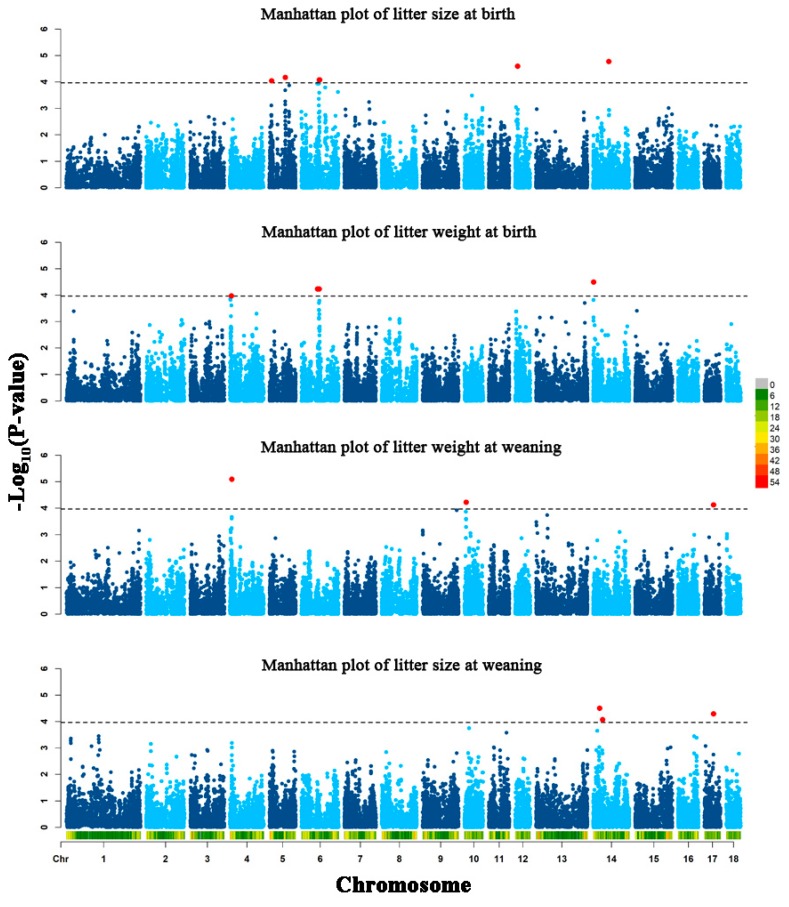
The Manhattan plots of four porcine reproductive traits distinguished by text labels. The y-axis of the Manhattan plots display the -log10 (*p*-value) of each SNP in the genome-wide association analysis. The black horizontal lines divide SNP with *p*-values <1.08e–4. The red dots stand for the potential SNPs associated with the traits of LSB, LWB, LSW, and LWW.

In total, 20 SNPs that reached the suggestive significance level were found to be associated with one of the tested reproductive traits (Table 3) and were defined as the potential SNPs for each trait. Manhattan plots were used to visualize the association results of the four traits (Figure 1). The most significant SNPs associated with LSB were rs80979042 and rs80825112, located in the intron of *BICC1* gene on chromosome 14. Additionally, the other five potential SNPs were located on chromosome 5, 6, and 12. For LWB, two SNPs located between 5.66 Mb and 5.68 Mb on chromosome 4, two SNPs located between 68.26 Mb and 74.78 Mb on chromosome 6, and a SNPs located on chromosome 14 at 1.18 Mb reached suggestive significance. For both LWW and LSW, SNP rs328230332 located in the intron of *FAM110A* on chromosome 17 was simultaneously associated with these two traits. Additionally, there were other four and two potential SNPs associated with LWW and LSW, respectively. By extending 1 Mb downstream and upstream of the potential SNPs, 146, 62, 58, and 63 functional genes were identified for LSB, LWB, LSW, and LWW, respectively. For LSB, the functional genes were enriched in “GO: 0061436, establishment of skin barrier”, “GO: 0045606, positive regulation of epidermal cell differentiation”, and some other epidermal growth-associated GO terms. For LWB, the functional genes were involved in organismal defense- and immunity-associated GO terms, including “GO: 0042742, defense response to bacterium” and “GO: 0045087, innate immune response”. Furthermore, the functional genes of LSW were enriched in “GO: 0042742, defense response to bacterium”, “GO: 0045087, innate immune response”, and “GO: 003511,~ embryonic forelimb morphogenesis”. Besides, the functional genes of LWW were involved in “GO: 0061436, establishment of skin barrier”, “GO: 0043552, positive regulation of phosphatidylinositol 3-kinase activity”, and “GO: 0034644, cellular response to UV” (Table 4). 

## 4. Discussion

In the present study, we used the Illumina Porcine SNP60 Chips and the GeneSeek GGP-Porcine Chip to genotype a Duroc pig population. Then, a genome-wide association analysis between the genotypes and four reproductive trait phenotypes was performed using a single-marker regression approach. Finally, 20 potential SNPs reaching suggestive significance were identified to be associated with the four pig reproductive traits. 

SNP rs328230332 was associated with both LWW and LSW. This SNP is located in the intron of the *FAM110A* gene (family with sequence similarity 110, member A). *FAM110A* has been reported to be significantly associated with young-onset hypertension in Han Chinese population of Taiwan [19]. In livestock, Gutiérrez-Gil et al. stated *FAM110A* is located in genomic regions associated with dairy production in sheep. SNP rs80979042 and rs80825112 on chromosome 14 were associated with LSB and are located within the intron of Bicaudal C Homolog 1 (*BICC1*) gene. *BICC1* has been identified by GWAS as a candidate gene associated with major depressive disorder in humans [20]. *BICC1* provokes renal and pancreatic cysts and the visceral left-right patterning of ectopic Wnt/β-catenin signaling during visceral left-right patterning [21]. In pigs, *BICC1* has been reported to be associated with prenatal development of skeletal muscles and to be expresses differently during myogenesis in Pietrain and Duroc pigs [22]. Two SNPs, rs81476258 and rs81326131, were annotated within the intron of *EPHB2*, which belongs to the Eph family, the largest known tyrosine kinase receptor (RTK) family [23]. *EPHB2* is activated by its ligand EphrinB1, which results in protein phosphorylation in cells. In embryonic development and postnatal life processes, *EPHB2* is expressed in a tissue-specific and time-specific manner [24] and also plays an important role in axon orientation, angiogenesis, and tumorigenesis [25]. Furthermore, *EPHB2* is involved in regulating postnatal myogenesis through inhibiting satellite cell formation in mice [26].

Additionally, several other candidate genes were identified to be potentially associated with these traits of interest. *KCNA1*, a member of potassium channels family, has been involved in the maturation of the mouse auditory forebrain [27]. *ZDHHC18* is the component of Hippo pathway, influencing skeletal muscle feed arteries in rat [28]. *FAM135B,* associated with the traits of LWB, has been related to mid-test metabolic weight in U.S. beef cattle [29]. *SEMA4D* differential expression has been reported in the skeletal muscle of pigs with distinct growth and fatness profiles [30]. For the functional enrichment analysis results, several biological processes GO terms were preferentially associated with the functional genes of more than two traits studied in the present research, including “GO: 0061436, establishment of skin barrier”, “GO: 0045087, innate immune response”, and “GO: 0042742, defense response to bacterium”. These results reveal that the functional genes detected in the present study, mainly involved in the growth of epidermal tissues, organismal defense, and immunity, are strongly associated with birth (alive or dead) and weaning in piglets [31]. There were no common SNPs between the present study and some previous studies [17,32], which might indicate that different pig breeds need different selection strategies for reproductive traits. 

Taking the DEBV as “dependent variable” could improve the power of GWAS. Generally, trait phenotype is the dependent variable in most GWAS. However, in this study, DEBVs obtained from on-farm genetic evaluation were employed, because they are more suitable than the original phenotypes. This is mainly because: (1) Systematic environmental effects are excluded from DEBVs; (2) More phenotypic records from relatives were combined to evaluate the individual under consideration; (3) Some trait phenotypes can be recorded only in one gender; and (4) They do not contain information from relatives. Recently, many GWAS have used DEBVs as “phenotype”, especially for livestock populations [33,34,35].

In this study, we have not identified significant SNPs associated with the four reproduction traits examined. We speculate that the relatively small number of SNPs reaching the suggestive significance level is partly due to the small size of the research population used in the present study. Additionally, the substructure within this population further decreased the effective population size and, hence, affected the power of GWAS. In a population with limited size, only large or moderate QTLs can be detected. Hence, research populations must be enlarged in our future studies to confirm the findings from the present study. 

## 5. Conclusions

Through a genome-wide association study of four reproductive traits in a Duroc pig herd, we detected 20 SNPs that were potentially associated with these traits of interest. *TXN2, KCNA1, ENSSSCG00000003546, ZDHHC18, MAP2K6, BICC1, FAM135B, EPHB2, SEMA4D, ST3GAL1, KCTD3, FAM110A, TMEM132D, TBX3,* and *FAM110A* might be important candidate genes that compose the underlying genetic architecture of porcine reproductive traits. These findings help to understand the genetic basis of porcine reproductive traits and could be potentially applied in pig breeding programs. 

## Figures and Tables

**Table 1:** Phenotypes (de-regressed estimated breeding values (EBVS)) of four porcine reproduction traits.

Trait ^a^	Mean	SD	N	Mean Reliability ^b^	h^2^
LSB	−0.015	0.526	1067	0.435	0.158
LWB	−0.061	0.099	1067	0.421	0.161
LWW	0.062	1.147	1067	0.511	0.173
LSW	1.604	6.454	1067	0.389	0.140

^a^: LSB = litter size at birth, LWB = litter weight at birth, LWW = litter size at weaning, LSW = litter weight at weaning; ^b^: reliability of EBV values.

**Table 2:** Genetic correlations between four reproduction traits.

Trait	LSB	LWB	LWW	LSW
LSB				
LWB	0.835 ± 0.037			
LWW	0.302 ± 0.127	0.500 ± 0.110		
LSW	0.509 ± 0.119	0.551 ± 0.115	0.880 ± 0.038	

**Table 3:** Potential single-nucleotide polymorphisms (SNPs) and candidate genes detected in the genome-wide association study for four porcine reproduction traits.

Traits	SNP	Chromosome	Position	*p*-Value	Allele Frequency	Allele Substitution Effect	Candidate Gene ^b^
LSB	rs336638152	5	9006723	9.09 × 10^−5^	0.398	−0.117	Thioredoxin 2 (*TXN2*)
	rs80999110	5	67782650	6.73 × 10^−5^	0.438	−0.118	Potassium voltage-gated channel subfamily A member 1 (*KCNA1*)
	rs81318862	6	76074229	8.29 × 10^−5^	0.109	−0.187	ENSSSCG00000003546
	rs329711941	6	77726418	8.28 × 10^−5^	0.107	−0.187	Zinc finger DHHC-type containing 18 (*ZDHHC18*)
	rs81439394	12	10118697	2.53 × 10^−5^	0.290	−0.141	Mitogen-activated protein kinase 6 (*MAP2K6*)
	rs80979042	14	66823174	1.69 × 10^−5^	0.479	−0.140	BICC1 (*BICC1*)
	rs80825112	14	66854605	1.69 × 10^−5^	0.479	−0.140	*BICC1*
LWB	rs325089329	4	5662747	1.06× 10^−4^	0.155	−0.345	Family with sequence similarity 135 member B (*FAM135B*)
	rs329734169	4	5677434	1.06× 10^−4^	0.155	−0.345	*FAM135B*
	rs81476258	6	68258924	5.85 × 10^−5^	0.407	0.276	EPH receptor B2 (*EPHB2*)
	rs81326131	6	74780466	5.85 × 10^−5^	0.407	0.276	*EPHB2*
	rs332491771	14	1176591	3.18 × 10^−5^	0.413	0.251	Semaphorin 4D (*SEMA4D*)
LWW	rs80808642	4	7289023	8.01 × 10^−6^	0.480	−1.629	ST3 beta-galactoside alpha-2,3-sialyltransferase 1 (*ST3GAL1*)
	rs81427863	10	7002238	5.98 × 10^−5^	0.215	−1.718	Potassium channel tetramerization domain containing 3 (*KCTD3*)
	rs322567083	10	7029718	5.98 × 10^−5^	0.215	−1.718	*KCTD3*
	rs81428034	10	7059506	5.98 × 10^−5^	0.215	−1.718	*KCTD3*
	rs328230332	17	39032680	7.50 × 10^−5^	0.100	2.338	Family with sequence similarity 110 member A (*FAM110A*)
LSW	rs339777110	14	27027253	3.12 × 10^−5^	0.168	0.032	Transmembrane protein 132D (*TMEM132D*)
	rs80947288	14	39275817	8.41 × 10^−5^	0.259	0.025	T-box 3 (*TBX3*)
	rs328230332	17	39032680	5.12 × 10^−5^	0.100	0.038	*FAM110A*

^b^: The SNP-containing or nearest annotated genes for each potential SNP.

**Table 4:** Biological process Gene Ontology (GO) terms enrichment analysis results.

Trait	Biological Process GO Terms
LSB	GO: 0061436, establishment of skin barrier; GO: 0045606, positive regulation of epidermal cell differentiation; GO: 0010482, regulation of epidermal cell division
LWB	GO: 0042742, defense response to bacterium; GO: 0045087, innate immune response
LWW	GO: 0061436, establishment of skin barrier; GO: 0043552, positive regulation of phosphatidylinositol 3-kinase activity; GO: 0034644, cellular response to UV
LSW	GO: 0042742, defense response to bacterium; GO: 0045087, innate immune response; GO: 0035115, embryonic forelimb morphogenesis
